# Calcium current properties in dystrophin‐deficient ventricular cardiomyocytes from aged mdx mice

**DOI:** 10.14814/phy2.13567

**Published:** 2018-01-15

**Authors:** Lena Rubi, Hannes Todt, Helmut Kubista, Xaver Koenig, Karlheinz Hilber

**Affiliations:** ^1^ Center for Physiology and Pharmacology Medical University of Vienna Vienna Austria

**Keywords:** Aging, Ca channel function, Ca transients, Duchenne muscular dystrophy, mdx mouse

## Abstract

Duchenne muscular dystrophy (DMD), caused by mutations in the gene encoding for the cytoskeletal protein dystrophin, is linked with severe cardiac complications including cardiomyopathy development and cardiac arrhythmias. We and others recently reported that currents through L‐type calcium (Ca) channels were significantly increased, and channel inactivation was reduced in dystrophin‐deficient ventricular cardiomyocytes derived from the mdx mouse, the most commonly used animal model for human DMD. These gain‐of‐function Ca channel abnormalities may enhance the risk of Ca‐dependent arrhythmias and cellular Ca overload in the dystrophic heart. All studies, which have so far investigated L‐type Ca channel properties in dystrophic cardiomyocytes, have used hearts from either neonatal or young adult mdx mice as cell source. In consequence, the dimension of the Ca channel abnormalities present in the severely‐diseased aged dystrophic heart has remained unknown. Here, we have studied potential abnormalities in Ca currents and intracellular Ca transients in ventricular cardiomyocytes derived from aged dystrophic mdx mice. We found that both the L‐type and T‐type Ca current properties of mdx cardiomyocytes were similar to those of myocytes derived from aged wild‐type mice. Accordingly, Ca release from the sarcoplasmic reticulum was normal in cardiomyocytes from aged mdx mice. This suggests that, irrespective of the presence of a pronounced cardiomyopathy in aged mdx mice, Ca currents and Ca release in dystrophic cardiomyocytes are normal. Finally, our data imply that dystrophin‐ regulation of L‐type Ca channel function in the heart is lost during aging.

## Introduction

Duchenne muscular dystrophy (DMD), induced by mutations in the gene encoding for the cytoplasmic protein dystrophin, is a severe inherited disease characterized by progressive muscle weakness and degeneration. Besides the relatively well‐characterized skeletal muscle pathology, DMD is also associated with cardiac complications. These include cardiomyopathy development and the occurrence of cardiac arrhythmias. The current understanding of the pathogenesis in the dystrophic heart is very limited, but recent research suggests that dysfunctional ion channels in cardiomyocytes play an important role. For example, we and others have shown that currents through L‐type calcium (Ca) channels were significantly increased (Koenig et al. 2014), and channel inactivation was reduced (Koenig et al. 2014; Sadeghi et al. 2002; Viola et al. 2014, 2013; Woolf et al. 2006) in dystrophic ventricular cardiomyocytes derived from mdx mice. The mdx mouse (Sicinski et al. 1989) is dystrophin‐deficient and the most commonly used mouse model of DMD. Both the named Ca channel abnormalities (increased current amplitudes and reduced inactivation) will increase the Ca influx into the dystrophic myocyte during an action potential (AP) and thereby enhance the risk of Ca‐dependent arrhythmias and cellular Ca overload (Zhou et al. 1998).

In all the above‐cited studies, which have investigated L‐type Ca channel properties in dystrophic cardiomyocytes, hearts from relatively young mdx mice (age range between only a few days‐old and 25 weeks) were used as cell source. This represents a major shortcoming for the following reason: The development of a distinct dilated cardiomyopathy (including functional impairments), comparable to that observed in DMD patients, takes at least 40 weeks in mdx mice (e.g., (Au et al. 2011; Quinlan et al. 2004; Sarma et al. 2010)). Consequently, the currently known “dystrophic Ca channel abnormalities” in mdx cardiomyocytes only reflect an early disease stage characterized by a mild or even missing cardiac phenotype. The more relevant dimension of the Ca channel abnormalities present in the severely impaired/diseased aged dystrophic heart, on the other hand, has remained unknown as yet.

Therefore, in this study, we have investigated the L‐type Ca current (*I*_Ca_) properties of dystrophic ventricular cardiomyocytes, which were derived from “aged” (≥1 year of age) mdx mice. At this age the mdx mouse model is known to exhibit a pronounced dilated cardiomyopathy accompanied by the occurrence of inflammation, cardiomyocyte necrosis, fibrosis, and contractile dysfunction (Au et al. 2011; Quinlan et al. 2004; Sarma et al. 2010). For comparison, the L‐type I_Ca_ properties of cardiomyocytes derived from wild‐type (wt) mice at the same age were studied. We hypothesized that cardiac ventricular Ca channel abnormalities are more severe in aged when compared with those previously described for younger dystrophic mice. Testing this hypothesis seemed reasonable because, in our previous study (Koenig et al. 2014), a significantly prolonged QT interval in the electrocardiogram (ECG) only occurred in aged mdx mice. Moreover, Ca overload was observed particularly in dystrophic cardiomyocytes from old but not young mdx mice (Mijares et al. 2014; Williams and Allen 2007). Besides L‐type I_Ca_, we have also studied T‐type *I*_Ca_ and intracellular Ca transients in ventricular cardiomyocytes derived from aged wt and mdx mice.

## Materials and Methods

### Ethical approval

The investigation conforms to the guiding principles of the Declaration of Helsinki and coincides with the rules of our University Animal Welfare Committee. The respective ethics vote for keeping and breeding dystrophic mice for organ withdrawal from killed animals has the following number: BMWFW‐66.009/0175‐WF/V/3b/2015.

### Mouse model and genotyping

Dystrophin‐ deficient mdx mice on the BL10 background (C57BL/10ScSn‐Dmdmdx/J), and respective wt control mice (C57BL/10ScSnJ) were purchased from Charles River Laboratories. Genotyping of the mice was performed using standard PCR‐assays.

### Isolation of ventricular cardiomyocytes

Male wt and mdx mice in an age range of 52–92 weeks (referred to as “aged mice” throughout the text) were killed by cervical dislocation. Cardiomyocytes were isolated from the ventricles of their hearts using a Langendorff setup as described in our previous work (Koenig et al. 2011).

### I_Ca_ recordings

Currents were recorded in the whole‐cell mode of the patch‐clamp technique from cardiomyocytes up to 8 h after preparation at an experimental temperature of 22 ± 1.5°C, using an Axoclamp 200B patch‐clamp amplifier (Axon Instruments, Union City, CA). Pipettes were pulled from aluminosilicate glass (AF150 –100‐10; Science Products, Hofheim, Germany) with a P‐97 horizontal puller (Sutter Instruments, Novato, CA), and had resistances between 1.0 and 1.8 MΩ when filled with pipette solution (see below). Data acquisition was performed with the pClamp 6.0 software (Axon Instruments) through a 12‐bit A‐D/D‐A interface (Digidata 1200; Axon Instruments). Data were low‐pass filtered with 2 kHz (3 dB) and digitized at 5 kHz. Leak currents and capacity transients were subtracted using a P/4 protocol. Data analyses were performed using Clampfit 10.2 (Axon Instruments) and GraphPad Prism 5.01 (San Diego, CA) software. The bath solution contained (in mmol/L) 10 CaCl_2_, 145 TEA‐Cl, 10 HEPES, pH 7.4 adjusted with TEA‐OH. The pipette solution consisted of 145 Cs‐aspartate, 2 MgCl_2_, 10 HEPES, 0.1 Cs‐EGTA, 2 Mg‐ATP, pH 7.4 adjusted with CsOH.

**Figure 1:**
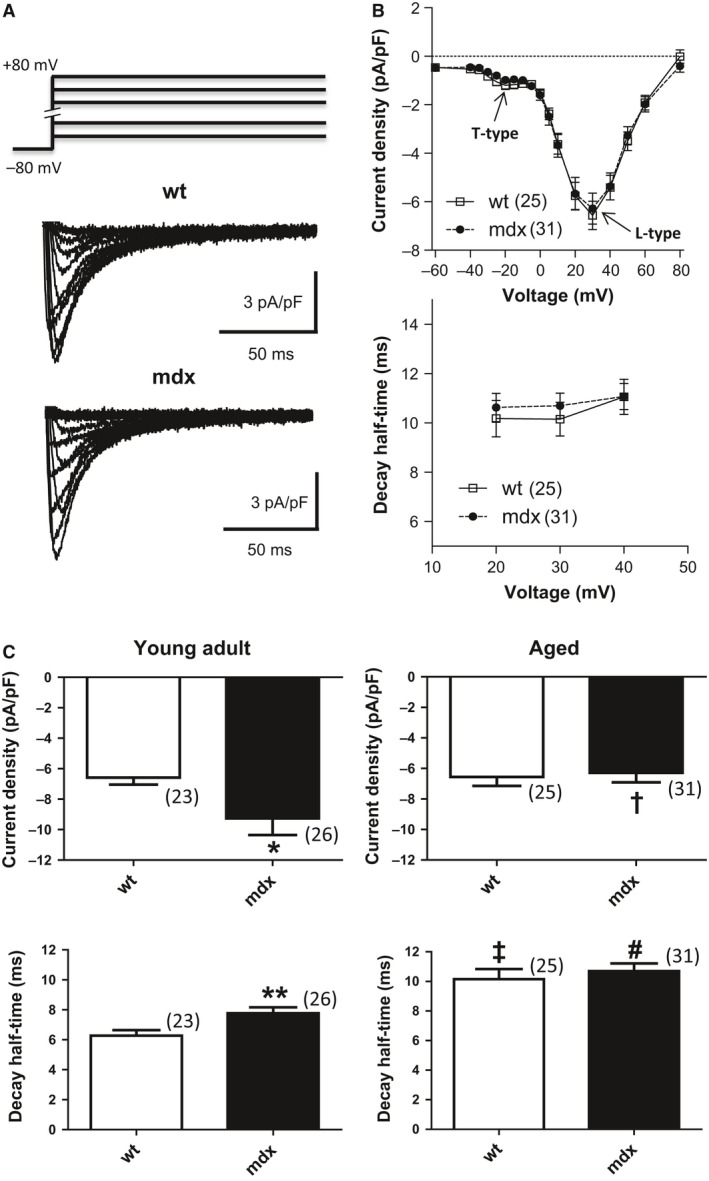
Ca current properties in wt and dystrophic (mdx) cardiomyocytes. (A) Original traces of Ca currents of a typical “aged” wt and mdx cardiomyocyte elicited by the pulse protocol displayed on top. (B) Current density‐voltage relationships (top) and current decay kinetics (bottom) of “aged” wt and mdx cardiomyocytes. The numbers of cells tested for each population (*n* numbers) are given in brackets. The current density‐voltage relations revealed two distinct negative peaks (indicated by arrows): one at −20 mV representative of T‐type Ca channel activation, and the other at +30 mV reflecting L‐type Ca channel activity. For comparison of the Ca current decay kinetics (bottom), decay half‐times (representing the time period between the current peak and the time point at which the current had decayed to 50%) were plotted against voltage. No significant differences between wt and mdx cardiomyocyte parameters were found. (C) Comparison of current density (top panel) and decay kinetics (lower panel) at +30 mV between “young adult” (left panel) and “aged” (right panel) wt and mdx cardiomyocytes. A test pulse potential of +30 mV was chosen because at this voltage L‐type Ca current was maximal. The respective n numbers are given in brackets. A significant number of the “young adult” cardiomyocyte data points were taken from our previous study (Koenig et al. 2014). *indicates a significant difference between the current density values of “young adult” wt and mdx cardiomyocytes with *P* < 0.05. **indicates a significant difference (*P* < 0.01) between the decay half‐times of “young adult” wt and mdx cells. †, decreased current densitiy in “aged” versus “young adult” mdx cells (*P* < 0.05) (top panel); ‡, increased decay half‐time in “aged” versus “young adult” wt cells (*P* < 0.01) (lower panel); #, increased decay half‐time in “aged” versus “young adult” mdx cells (*P* < 0.01) (lower panel).

The currents were elicited from a holding potential of −80 mV by depolarizing voltage steps up to +80 mV (see Fig. 1A, top). For the determination of current density‐voltage relations, the current amplitudes at various voltages were measured. These were then divided by the cell capacitance to obtain current densities, which were plotted against the respective test pulse potentials. Current density‐voltage relations revealed two distinct negative peaks (see Fig. 1B, top): a small one at ‐20 mV representative of T‐type Ca channel activation, and a large one at +30 mV reflecting L‐type Ca channel activity. The kinetics of L‐type *I*_Ca_ inactivation (Fig. 1B, bottom) was analyzed by measuring the time period between the current peak, and the time point at which the current had decayed to 50%. This parameter, also used in our previous studies (Koenig et al. 2011, 2014), is termed “decay half‐time.”

### Confocal Ca imaging

Isolated cardiomyocytes were seeded on 3.5 cm glass bottom cell culture dishes. A minimum of 1 h was allowed for cell attachment. Cardiomyocytes were then treated with 5 *μ*mol/L of the cell membrane permeable Ca‐ sensitive fluorescent dye fluo‐4 AM (Thermo Fisher Scientific, Vienna, Austria) and incubated for 20 min at 37°C and 5 % CO_2_. Thereafter the medium was removed and myocytes were bathed in an extracellular solution containing (in mmol/L): 140 NaCl, 4 KCl, 2 CaCl_2_, 2 MgCl_2_, 5 HEPES, 5 Glucose, pH adjusted to 7.4 with NaOH. A quantity of 20 *μ*mol/L of the myosin inhibitor blebbistatin was added to inhibit contractions. A time period of 20 min was allowed for de‐esterification of the dye. Two parallel platinum electrodes were inserted into the dish to allow for electrical stimulation delivered by a Grass Square Pulse Stimulator (S88). Single rectangular pulses of 2–4 msec duration were applied with an amplitude of 40 V. A superfusion manifold was positioned in the immediate vicinity of the cell connected to an air pressure driven OctaFlow II perfusion system (ALA Scientific Instruments, Westbury, NY). Cells were continuously superfused with external solution at a rate of 2 *μ*L sec^‐1^. To elicit caffeine‐induced sarcoplasmic reticulum Ca release, superfusion was switched to external solution containing 10 mmol caffeine.

Glass bottom culture dishes were mounted above a 20x air objective (NA 0.75) of a confocal microscope system (Nikon A1R+) equipped with a 12 kHz resonant scanner. Images were acquired in confocal mode with the pinhole set to between 2 and 5 airy units. A 488 nm Argon laser line at an intensity of 0.5–2% was used for excitation. Emitted dye fluorescence was collected with a single bandpass filter (525/50 nm). xyt image series were acquired at a sample rate of 90 msec frame^−1^. Collected dye fluorescence was averaged over a region of interest from within the physical dimension of the cardiomyocyte, and background fluorescence was subtracted to monitor fluorescence over time, F(t). Only quiescent myocytes were selected for experiments. These were first electrically stimulated with a 30 sec train of 0.1 Hz. Thereafter the myocytes were allowed to recover for 60 sec before being challenged with 10 mmol/L caffeine for 30 sec. Fluorescent peaks upon stimulation with single electrical pulses or with caffeine were evaluated relative to baseline fluorescence prior to stimulation (F0). To evaluate the duration of the elicited Ca transients, a single exponential function was fit to the decaying fluorescence to obtain respective time constants (tau values).

### Statistical analyses

Data are expressed as means ± SE. Statistical comparisons between wt and mdx cardiomyocytes, or “young adult” (derived from 15 to 25 week‐old mice) and “aged” (derived from 52 to 92 week‐old mice) myocytes were made using an unpaired two‐tailed Student's *t*‐test. A *P* < 0.05 was considered significant.

## Results

### Normal L‐type Ca channel function in “aged” dystrophic (mdx) cardiomyocytes

Figure 1A shows typical original traces of I_Ca_ recorded from a normal (wt) and dystrophic (mdx) cardiomyocyte, which were derived from an aged wt and mdx mouse, respectively. The currents were elicited by the pulse protocol displayed on top. A summary of the current density‐voltage relationships, derived from a series of such experiments, is presented in Figure 1B (top). It can be noticed that the current densities of wt and dystrophic cardiomyocytes were similar over a wide range of tested potentials. This suggests that dystrophin deficiency does not affect the I_Ca_ density in cardiomyocytes derived from aged mice. Analysis of the current decay after channel activation (Fig. 1B, bottom) revealed that the kinetics of inactivation was also comparable in wt and mdx cardiomyocytes. These data imply that, similar to current density in myocytes of aged mice, the kinetics of channel inactivation is independent of the expression of dystrophin.

To allow for assessment of age‐related changes in Ca channel properties, the current density and decay values at +30 mV (taken from Fig 1B) were related to the respective parameters we previously detected in cardiomyocytes derived from young adult (15–25 weeks of age) wt and mdx mice (Koenig et al. 2014) in Figure. 1C. It can be observed that current density (increased in “young adult” but not “aged” mdx cardiomyocytes when compared to age‐matched wt control) was not affected by aging in wt but reduced in mdx cells (Fig. 1C, top panel). Moreover, aging slowed the kinetics of inactivation both in wt and mdx cardiomyocytes, whereby this effect was more pronounced in wt cells (Fig. 1C, lower panel). Together the data suggest that aging has a different impact on the Ca channel properties in wt and dystrophic hearts, finally resulting in similar properties at over 1 year of animal age.

### T‐type I_Ca_ is not upregulated in “aged” mdx cardiomyocytes

Although only slightly present in healthy adult cardiac ventricles, increased ventricular re‐expression of T‐type Ca channels may contribute to the progression of heart failure (Kinoshita et al. 2009; Vassort et al. 2006). Thus, it is possible that T‐type I_Ca_ is enhanced in “aged” dystrophic ventricular cardiomyocytes, which may lead to abnormally increased Ca influx into dystrophic cells. Figure 1B (top) shows that we could only detect very tiny T‐type I_Ca_ (see arrow “T‐type”) both in “aged” wt and mdx cardiomyocytes. Differences in current density or voltage‐dependence of channel activation between normal and dystrophic cells were not detectable (Fig. 1B), and activation as well as inactivation kinetics of the currents appeared similar. This suggests that T‐type *I*_Ca_ is neither upregulated nor dysregulated in “aged” dystrophic cardiomyocytes.

### Intracellular Ca transients in “aged” wt and mdx cardiomyocytes

**Figure 2:**
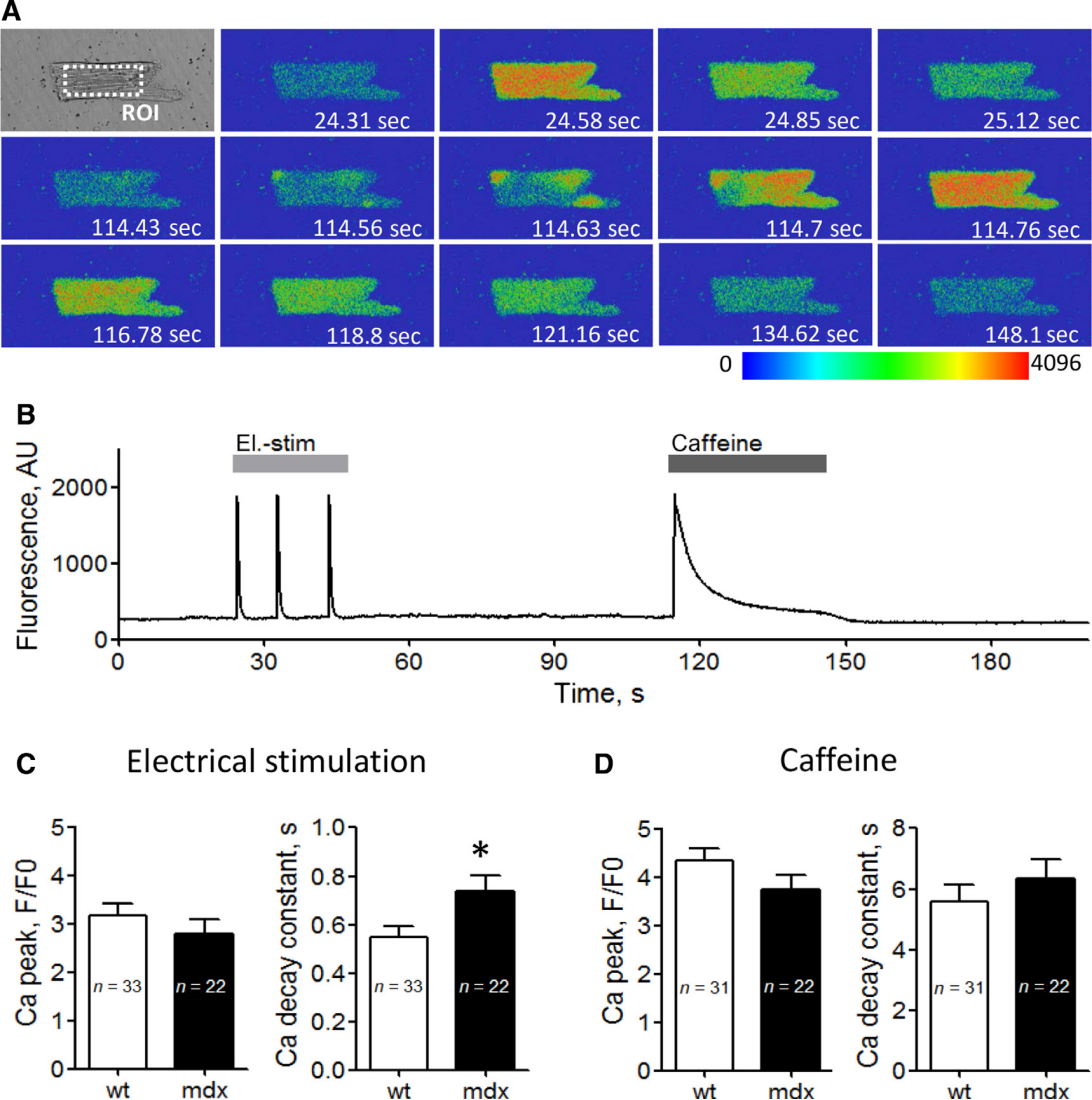
Electrically‐ and caffeine‐ induced cytosolic Ca transients in wt and dystrophic (mdx) cardiomyocytes. (A) Representative transmitted light image (top left) and image series of false color Fluo‐4 fluorescence during the course of a typical experiment as shown in (B) xyt image series was acquired with a sampling rate of 90 msec frame^−1^, and emitted Fluo‐4 fluorescence was spatially averaged from a region of interest (ROI) drawn in the central region of a cardiomyocyte. (B) Time course of averaged Fluo‐4 fluorescence reporting rises in cytosolic Ca concentration during electrical field stimulation (el.‐stim, light gray bar) and application of 10 mmol/L caffeine (dark gray bar). AU, arbitrary units. (C) Mean Ca peak fluorescence relative to baseline (F/F0) (left), and Ca transient decay constant (right) elicited by electrical field stimulation for “aged” wt and dystrophic (mdx) cardiomyocytes. (D) Mean Ca peak fluorescence relative to baseline (F/F0) (left) and Ca transient decay constant (right) elicited by application of 10 mmol/L of the ryanodine receptor agonist caffeine for aged wt and mdx cardiomyocytes. The respective numbers of cells tested (*n*) are given within the bars. * indicates a significant difference in the el.‐stim induced Ca transient decay between wt and mdx with *P* < 0.05. No other statistically significant differences were found.

During the plateau phase of the cardiac AP, Ca influx through L‐type Ca channels into the cell triggers Ca‐induced Ca release from the sarcoplasmic reticulum, which finally initiates contraction. To check if normal L‐type Ca channel function in “aged” mdx cardiomyocytes goes along with normal Ca release, we compared electrically evoked Ca transients between wt and mdx myocytes derived from aged mice (Fig. 2). Figure 2C (left) shows that the peak of the Ca signal was similar in wt and mdx cells, suggesting normal Ca release in “aged” dystrophic cardiomyocytes. The decay of the Ca signal after electrical stimulation, on the other hand, was significantly slowed in mdx myocytes (Fig. 2C, right). Finally, when caffeine was used to elicit Ca transients (Fig. 2D), both the peak of the Ca signal (left) and the decay of the Ca transient (right) were similar in “aged” wt and mdx cardiomyocytes.

## Discussion

### Age‐related Ca channel functional changes in wt cardiomyocytes

In senescent cardiomyocytes, the amplitude of currents through L‐type Ca channels is significantly increased. Because this typically occurs in parallel with an enlargement of the myocytes, the I_Ca_ density (current per membrane surface) remains unaltered (see (Feridooni et al. 2015; Li et al. 2014; Zhou et al. 1998)). Furthermore, Ca channel inactivation is slowed in “aged” cardiomyocytes, resulting in an increased net Ca influx during each AP in senescent compared to young hearts. On one hand, this augmentation of Ca influx may provide a compensatory mechanism to preserve cardiac function in the senescent heart in the basal state. On the other hand, enhanced Ca influx also raises the risk of Ca overload and Ca‐dependent arrhythmias (Zhou et al. 1998). Comparison of the I_Ca_ properties from “aged” wt cardiomyocytes (present study) with the data from our previous study (Koenig et al. 2014) revealed unaltered current densities but markedly slowed inactivation in “aged” compared with “young adult” wt cardiomyocytes (Fig. 1C). These findings are well consistent with previous reports (see above; (Feridooni et al. 2015; Li et al. 2014; Zhou et al. 1998)). Interestingly, the established age‐related changes in L‐type Ca channel function in wt cardiomyocytes are similar to Ca channel adaptations occurring in myocytes of diseased (hypertrophied or failing) hearts (see (Zhou et al. 1998) and references therein). This suggests that the aging process *per se* induces disadvantageous adaptations in cardiac Ca channel function.

### Normal Ca channel function in “aged” dystrophic cardiomyocytes

Gain of function L‐type Ca channel abnormalities in dystrophic cardiomyocytes (enhanced current density and impaired inactivation) are a hallmark of the disease phenotype in the dystrophic heart when studied in young dystrophic mice (between a few days and 25 weeks of age) (Koenig et al. 2014; Sadeghi et al. 2002; Viola et al. 2014, 2013; Woolf et al. 2006). In this study, to our surprise, we found that the I_Ca_ properties of dystrophic cardiomyocytes derived from aged mdx mice were similar to those of myocytes derived from wt mice in the respective age. This implies that, irrespective of the presence of a pronounced dilated cardiomyopathy in aged mdx mice (Au et al. 2011; Quinlan et al. 2004; Sarma et al. 2010), the I_Ca_ properties in dystrophic cardiomyocytes are normal. Furthermore, the data suggest that dystrophin‐ regulation of L‐type Ca channel function in the heart is completely lost during the murine aging process. Possibly, this can be explained by a significant loss of dystrophin protein in the senescent murine heart (Townsend et al. 2011). Besides the present study, to the best of our knowledge, only (Li et al. 2014) have so far investigated L‐ type Ca channel properties in “aged” dystrophic ventricular cardiomyocytes (derived from mdx mice). These authors reported increased current densities in cardiomyocytes derived from 12‐month‐old mdx compared to age‐matched wt mice. The apparent difference to our results (similar current densities in “aged” wt and mdx cardiomyocytes) may be explained by two circumstances: (1) the use of a considerably older mouse population in this study; (2) the use of male mice only (present study) versus both male and female animals in (Li et al. 2014). In contrast to our article, Ca channel inactivation was not studied in (Li et al. 2014), because the *I*_Ca_ recordings were only a secondary aspect of this investigation.

Our study further suggests that normal Ca channel function in “aged” dystrophic cardiomyocytes goes along with normal intracellular Ca release. This can be deduced from similar electrically evoked Ca transient peaks in cardiomyocytes derived from aged wt and mdx mice, in agreement with a respective finding in (Li et al. 2014). Ca release induced by caffeine application was also similar in “aged” wt and mdx cardiomyocytes. The significantly slowed decay of the Ca signal triggered by electrical stimulation in “aged” mdx myocytes we report coincides with similar findings in the literature (e.g., (Gonzalez et al. 2014; Williams and Allen 2007)), and can be explained by impaired Ca removal from the cytosol after release in dystrophic cells (Williams and Allen 2007).

As opposed to similar *I*_Ca_ properties in “aged” wt and dystrophic cardiomyocytes under basal conditions (present study), a clear cut difference between wt and mdx was previously reported for Ca channel responsiveness to beta‐ adrenergic stimuli. Thus, I_Ca_ enhancement by isoprenaline, as observed in “aged” wt cardiomyocytes, was almost completely lacking in “aged” mdx cells (Li et al. 2014). Reduced Ca channel responsiveness to beta‐adrenergic stimuli in “aged” mdx cardiomyocytes may be caused by a marked age‐related deterioration in *β*_1_‐adrenoceptor function (Lu and Hoey 2000). Irrespective of normal basal *I*_Ca_ properties in “aged” dystrophic cardiomyocytes, this represents a potential cause of reduced functionality and abnormal electrical properties in the aged dystrophic heart.

Shortened PQ and prolonged QTc intervals are hallmarks of the ECG in old mdx mice (Bostick et al. 2010; Koenig et al. 2014; Pereira et al. 2017). According to our finding of similar I_Ca_ properties in “aged” wt and mdx cardiomyocytes, we propose that other mechanisms than Ca channel abnormalities must generate these ECG deviations (and potentially associated cardiac arrhythmias (Colussi et al. 2010; Fauconnier et al. 2010; Gonzalez et al. 2015)) in mdx mice. Among various potential candidates are abnormal connexin 40 (Cx40) expression and impaired I_K1_ potassium channel function. First, consistent with a prolonged PQ interval in Cx40^−/−^ mice (Simon et al. 1998), significantly increased Cx40 protein levels in the mdx heart (Colussi et al. 2010) may contribute to the shortened PQ interval observed in ECGs from old mdx mice. Second, a decreased inward rectifier potassium current I_K1_ in dystrophic ventricular cardiomyocytes (Rubi et al. 2017) may prolong the QTc interval in the “dystrophic ECG”. These as well as potential other mechanistic considerations, however, remain speculative at present.

Finally, as gain of function Ca channel abnormalities do not exist in “aged” dystrophic (mdx) cardiomyocytes, they can also not account for the Ca overload, which was observed particularly in dystrophic cardiomyocytes from old mdx mice (Mijares et al. 2014; Williams and Allen 2007).

We conclude that, irrespective of the presence of a pronounced cardiomyopathy in aged mdx mice, Ca currents and Ca release in dystrophic cardiomyocytes are normal. Moreover, dystrophin‐ regulation of L‐type Ca channel function in the heart is lost during aging.

## Conflict of Interest

No conflicts of interest, financial or otherwise, are declared by the author(s).

## References

[phy213567-bib-0001] Au, C. G. , T. L. Butler , M. C. Sherwood , J. R. Egan , K. N. North , and D. S. Winlaw . 2011 Increased connective tissue growth factor associated with cardiac fibrosis in the mdx mouse model of dystrophic cardiomyopathy. Int. J. Exp. Pathol. 92:57–65. 21121985 10.1111/j.1365-2613.2010.00750.x PMC3052757

[phy213567-bib-0002] Bostick, B. , Y. Yue , and D. Duan . 2010 Gender influences cardiac function in the mdx model of Duchenne cardiomyopathy. Muscle Nerve 42:600–603. 20878741 10.1002/mus.21763 PMC3109082

[phy213567-bib-0003] Colussi, C. , R. Berni , J. Rosati , S. Straino , S. Vitale , F. Spallotta , et al. 2010 The histone deacetylase inhibitor suberoylanilide hydroxamic acid reduces cardiac arrhythmias in dystrophic mice. Cardiovasc. Res. 87:73–82. 20164117 10.1093/cvr/cvq035

[phy213567-bib-0004] Fauconnier, J. , J. Thireau , S. Reiken , C. Cassan , S. Richard , S. Matecki , et al. 2010 Leaky RyR2 trigger ventricular arrhythmias in Duchenne muscular dystrophy. Proc. Natl Acad. Sci. USA 107:1559–1564. 20080623 10.1073/pnas.0908540107 PMC2824377

[phy213567-bib-0005] Feridooni, H. A. , K. M. Dibb , and S. E. Howlett . 2015 How cardiomyocyte excitation, calcium release and contraction become altered with age. J. Mol. Cell. Cardiol. 83:62–72. 25498213 10.1016/j.yjmcc.2014.12.004

[phy213567-bib-0006] Gonzalez, D. R. , A. V. Treuer , G. Lamirault , V. Mayo , Y. Cao , R. A. Dulce , et al. 2014 NADPH oxidase‐2 inhibition restores contractility and intracellular calcium handling and reduces arrhythmogenicity in dystrophic cardiomyopathy. Am. J. Physiol. Heart Circ. Physiol. 307:H710–H721. 25015966 10.1152/ajpheart.00890.2013 PMC4187396

[phy213567-bib-0007] Gonzalez, J. P. , J. Ramachandran , L. H. Xie , J. E. Contreras , and D. Fraidenraich . 2015 Selective Connexin43 Inhibition Prevents Isoproterenol‐Induced Arrhythmias and Lethality in Muscular Dystrophy Mice. Sci. Rep. 5:13490. 26311238 10.1038/srep13490 PMC4550874

[phy213567-bib-0008] Kinoshita, H. , K. Kuwahara , M. Takano , Y. Arai , Y. Kuwabara , S. Yasuno , et al. 2009 T‐type Ca2 + channel blockade prevents sudden death in mice with heart failure. Circulation 120:743–752. 19687356 10.1161/CIRCULATIONAHA.109.857011

[phy213567-bib-0009] Koenig, X. , S. Dysek , S. Kimbacher , A. K. Mike , R. Cervenka , P. Lukacs , et al. 2011 Voltage‐gated ion channel dysfunction precedes cardiomyopathy development in the dystrophic heart. PLoS ONE 6:e20300. 21677768 10.1371/journal.pone.0020300 PMC3100353

[phy213567-bib-0010] Koenig, X. , L. Rubi , G. J. Obermair , R. Cervenka , X. B. Dang , P. Lukacs , et al. 2014 Enhanced currents through L‐type calcium channels in cardiomyocytes disturb the electrophysiology of the dystrophic heart. Am. J. Physiol. Heart Circ. Physiol. 306:H564–H573. 24337461 10.1152/ajpheart.00441.2013 PMC4892346

[phy213567-bib-0011] Li, Y. , S. Zhang , X. Zhang , J. Li , X. Ai , L. Zhang , et al. 2014 Blunted cardiac beta‐adrenergic response as an early indication of cardiac dysfunction in Duchenne muscular dystrophy. Cardiovasc. Res. 103:60–71. 24812281 10.1093/cvr/cvu119 PMC4133593

[phy213567-bib-0012] Lu, S. , and A. Hoey . 2000 Age‐ and sex‐associated changes in cardiac beta 1‐adrenoceptors from the muscular dystrophy (mdx) mouse. J. Mol. Cell. Cardiol. 32:1661–1668. 10966828 10.1006/jmcc.2000.1200

[phy213567-bib-0013] Mijares, A. , F. Altamirano , J. Kolster , J. A. Adams , and J. R. Lopez . 2014 Age‐dependent changes in diastolic Ca(2 + ) and Na(+) concentrations in dystrophic cardiomyopathy: role of Ca(2 + ) entry and IP3. Biochem. Biophys. Res. Commun. 452:1054–1059. 25242522 10.1016/j.bbrc.2014.09.045 PMC4275309

[phy213567-bib-0014] Pereira, J. A. , A. F. Mauricio , M. J. Marques , and H. S. Neto . 2017 Dual Therapy Deflazacort/Doxycyclyne Is Better Than Deflazacort Monotherapy to Alleviate Cardiomyopathy in Dystrophin‐Deficient mdx Mice. J. Cardiovasc. Pharmacol. Ther. 22:458–466. 28793824 10.1177/1074248416686189

[phy213567-bib-0015] Quinlan, J. G. , H. S. Hahn , B. L. Wong , J. N. Lorenz , A. S. Wenisch , and L. S. Levin . 2004 Evolution of the mdx mouse cardiomyopathy: physiological and morphological findings. Neuromuscul. Disord. 14:491–496. 15336690 10.1016/j.nmd.2004.04.007

[phy213567-bib-0016] Rubi, L. , X. Koenig , H. Kubista , H. Todt , and K. Hilber . 2017 Decreased inward rectifier potassium current IK1 in dystrophin‐deficient ventricular cardiomyocytes. Channels (Austin) 11:101–108. 27560040 10.1080/19336950.2016.1228498 PMC5398571

[phy213567-bib-0017] Sadeghi, A. , A. D. Doyle , and B. D. Johnson . 2002 Regulation of the cardiac L‐type Ca2 + channel by the actin‐binding proteins alpha‐actinin and dystrophin. Am. J. Physiol. Cell Physiol. 282:C1502–C1511. 11997265 10.1152/ajpcell.00435.2001

[phy213567-bib-0018] Sarma, S. , N. Li , R. J. van Oort , C. Reynolds , D. G. Skapura , and X. H. Wehrens . 2010 Genetic inhibition of PKA phosphorylation of RyR2 prevents dystrophic cardiomyopathy. Proc. Natl Acad. Sci. USA 107:13165–13170. 20615971 10.1073/pnas.1004509107 PMC2919918

[phy213567-bib-0019] Sicinski, P. , Y. Geng , A. S. Ryder‐Cook , E. A. Barnard , M. G. Darlison , and P. J. Barnard . 1989 The molecular basis of muscular dystrophy in the mdx mouse: a point mutation. Science 244:1578–1580. 2662404 10.1126/science.2662404

[phy213567-bib-0020] Simon, A. M. , D. A. Goodenough , and D. L. Paul . 1998 Mice lacking connexin 40 have cardiac conduction abnormalities characteristic of atrioventricular block and bundle branch block. Curr. Biol. 8:295–298. 9501069 10.1016/s0960-9822(98)70113-7

[phy213567-bib-0021] Townsend, D. , M. Daly , J. S. Chamberlain , and J. M. Metzger . 2011 Age‐dependent dystrophin loss and genetic reconstitution establish a molecular link between dystrophin and heart performance during aging. Mol. Ther. 19:1821–1825. 21730971 10.1038/mt.2011.120 PMC3188736

[phy213567-bib-0022] Vassort, G. , K. Talavera , and J. L. Alvarez . 2006 Role of T‐type Ca^2+^ channels in the heart. Cell Calcium 40:205–220. 16766028 10.1016/j.ceca.2006.04.025

[phy213567-bib-0023] Viola, H. M. , S. M. Davies , A. Filipovska , and L. C. Hool . 2013 L‐type Ca2 + channel contributes to alterations in mitochondrial calcium handling in the mdx ventricular myocyte. Am. J. Physiol. Heart Circ. Physiol. 304:H767–H775. 23335798 10.1152/ajpheart.00700.2012

[phy213567-bib-0024] Viola, H. M. , A. M. Adams , S. M. Davies , S. Fletcher , A. Filipovska , and L. C. Hool . 2014 Impaired functional communication between the L‐type calcium channel and mitochondria contributes to metabolic inhibition in the mdx heart. Proc. Natl Acad. Sci. USA 111:E2905–E2914. 24969422 10.1073/pnas.1402544111 PMC4104855

[phy213567-bib-0025] Williams, I. A. , and D. G. Allen . 2007 Intracellular calcium handling in ventricular myocytes from mdx mice. Am. J. Physiol. Heart Circ. Physiol. 292:H846–H855. 17012353 10.1152/ajpheart.00688.2006

[phy213567-bib-0026] Woolf, P. J. , S. Lu , R. Cornford‐Nairn , M. Watson , X. H. Xiao , S. M. Holroyd , et al. 2006 Alterations in dihydropyridine receptors in dystrophin‐deficient cardiac muscle. Am. J. Physiol. Heart Circ. Physiol. 290:H2439–H2445. 16415078 10.1152/ajpheart.00844.2005

[phy213567-bib-0027] Zhou, Y. Y. , E. G. Lakatta , and R. P. Xiao . 1998 Age‐associated alterations in calcium current and its modulation in cardiac myocytes. Drugs Aging 13:159–171. 9739504 10.2165/00002512-199813020-00007

